# Effectiveness and cost-effectiveness of a health coaching intervention to improve the lifestyle of patients with knee osteoarthritis: cluster randomized clinical trial

**DOI:** 10.1186/s12891-015-0501-x

**Published:** 2015-02-25

**Authors:** Victoria Carmona-Terés, Iris Lumillo-Gutiérrez, Lina Jodar-Fernández, Teresa Rodriguez-Blanco, Joanna Moix-Queraltó, Enriqueta Pujol-Ribera, Xavier Mas, Enrique Batlle-Gualda, Milena Gobbo-Montoya, Anna Berenguera

**Affiliations:** Departamento de Psicología Básica, Universitat Autònoma de Barcelona; Facultad de Psicología, evolutiva y de la Salud. Edificio B. Campus de la UAB, Bellaterra, 08193 Barcelona, Spain; Centro Atención Primaria Can Bou, Calle Ciutat de Màlaga, 18-20, Castelldefels, 08860 Barcelona, Spain; Centro Atención Primaria Sant Ildefons, Avda República Argentiana s/n, Cornellà de Llobregat, 08940 Barcelona, Spain; Institut Universitari d’Investigació en Atenció Primària (IDIAP Jordi Gol), Gran Via Corts Catalanes, 587, àtic, Barcelona, 08007 Spain; Universitat Autònoma de Barcelona, Bellaterra (Cerdanyola del Vallès), Spain; Centro de Atención Primaria Amadeu Torner, Calle Amadeu Torner, 63, l’Hospitalet de Llobregat, 08902 Barcelona, Spain; Hospital Universitario de San Juan de Alicante; Unidad de Reumatología, Ctra N-332, Sant Joan d’Alacant, Alicante-Valencia, 03550 Spain; Psicología del dolor y en enfermedades reumáticas, Av. Presidente Carmona, 10 bis 1ºA, Madrid, 28020 Spain

**Keywords:** Knee osteoarthritis, Cluster randomized clinical trial, Qualitative research, Primary health care

## Abstract

**Background:**

The prevalence of osteoarthritis and knee osteoarthritis in the Spanish population is estimated at 17% and 10.2%, respectively. The clinical guidelines concur that the first line treatment for knee osteoarthritis should be non-pharmacological and include weight loss, physical activity and self-management of pain. Health Coaching has been defined as an intervention that facilitates the achievement of health improvement goals, the reduction of unhealthy lifestyles, the improvement of self-management for chronic conditions and quality of life enhancement.

The aim of this study is to analyze the effectiveness, cost-effectiveness and cost-utility of a health coaching intervention on quality of life, pain, overweight and physical activity in patients from 18 primary care centres of Barcelona with knee osteoarthritis.

**Methods/Design:**

Methodology from the Medical Research Council on developing complex interventions. **Phase 1:** Intervention modelling and operationalization through a qualitative, socioconstructivist study using theoretical sampling with 10 in-depth interviews to patients with knee osteoarthritis and 4 discussion groups of 8–12 primary care professionals, evaluated using a sociological discourse analysis. **Phase 2:** Effectiveness, cost-effectiveness and cost-utility study with a community-based randomized clinical trial. *Participants*: 360 patients with knee osteoarthritis (180 in each group). *Randomization unit*: Primary Care Centre. *Intervention Group*: will receive standard care plus 20-hour health coaching and follow-up sessions. *Control Group*: will receive standard care. *Main Outcome Variable*: quality of life as measured by the WOMAC index. *Data Analyses*: will include standardized response mean and multilevel analysis of repeated measures. *Economic analysis:* based on cost-effectiveness and cost-utility measures. **Phase 3:** Evaluation of the intervention programme with a qualitative study. Methodology as in Phase 1.

**Discussion:**

If the analyses show the cost-effectiveness and cost-utility of the intervention the results can be incorporated into the clinical guidelines for the management of knee osteoarthritis in primary care.

**Trial registration:**

ISRCTN57405925. Registred 20 June 2014.

## Background

Knee osteoarthritis (KO) is a chronic condition characterized by the progressive deterioration of articular cartilage and subchondral bone. KO has multiple causes that produce similar biological, morphological and clinical symptoms. The major clinical features of KO are pain with physical activity, restricted ability to walk and stand, and a progressive deformation of the knee joint. KO is frequently accompanied by obesity/overweight (90%), hypertension (40%), depression (30%) and diabetes (15%). These comorbidities contribute to a decreased quality of life [1].

Globally, it is estimated that 24% of the general adult population suffers from osteoarthritis. Symptomatic osteoarthritis affects 9.6% men and 18% women 60 years or older and its prevalence increases with age, up to 80% in people over 65 years of age in high-income countries. The increase in life expectancy will raise the number of patients affected by osteoarthritis. In the USA, 19% of the general population has a radiographic diagnosis of KO and 7% present knee symptoms. The prevalence of osteoarthritis is 17% and 16.6% in the general Spanish and Catalan populations, respectively, while the prevalence of knee osteoarthritis is 10.2% [2].

Osteoarthritis is responsible for the loss of 1.9 Quality Adjusted Life Years (QALYs) in people between 50 and 84 years of age [3]. The loss in obese individuals can reach 3.5 QALYs. According to Hunter [1], mortality rates in patients with osteoarthritis are higher compared with the general population (Standardized Mortality Ratio 1.55: 95% CI: 1.41-1.79). Osteoarthritis generates high costs (€1,502 per patient yearly), most of which (86%) are direct costs derived from medical care and dependency, such as help at home and at work. Indirect costs refer to loss of productivity and include help at home for affected housewives. It is, therefore, a condition with a high social impact [4]. Studies carried out in Norway and Spain show that the national cost for osteoarthritis can reach €3,528 million [5] and €4,700 million [4], respectively.

Clinical guidelines for osteoarthritis recommend a non-pharmacological first-line treatment for patients with osteoarthritis which should include weight loss, healthy eating habits, physical activity, self-management of pain, information-education and orthoses [6]. In particular, they recommend the education of patients even if the current evidence remains inconclusive [2,7,8]. Indeed, non-pharmacological recommendations frequently lack precision regarding contents, duration, intensity and frequency and might result in the suboptimal care afforded to osteoarthritis patients observed in several studies [2].

A great diversity of educational interventions takes place in primary care. Most of them are based on brief advice as recommended in the clinical guidelines and in primary care journals. Accordingly, the recommendations related to osteoarthritis within the “*Health Plan for Rheumatological and Musculoskeletal Conditions*” [9] aim to encourage all measures that enhance quality of life through health promotion, prevention and self-care, with a particular emphasis on modifiable risk factors such as obesity, optimal use of orthoses and exercise. The recommendations also underscore the need to minimize variability in clinical outcomes by improving the efficiency of diagnosis and therapy through a specific training of primary care doctors and nurses.

Self-management education programmes targeting patient education and behaviour modification were not more effective than usual clinical practice, information or other alternative therapies according to Kroon et al. [10]. However, the authors conclude that clinical trials that assess other self-management educational programmes for osteoarthritis might be warranted. Our study is based on the behaviour change framework [11], which takes into account Argyris’ theory [12] and the links between change processes and determinants of behaviour such as Prochaska’s stages of change [13].

Some interventions that focus on the promotion of healthy habits include two essential aspects: complexity and use of health coaching. The complex, multimodal and multidisciplinary design results in an intervention better suited to its context which therefore achieves better results and greater sustainability. The Medical Research Council has established a methodology for complex interventions that consists of several phases that can be repeatedly implemented and that use qualitative and quantitative methods [14].

Health Coaching originates within the conceptual framework of behaviour change and specifically in Argyris’ Action Theory [12]. Health Coaching is a behavioural intervention to facilitate patients adopt and sustain their own health related goals, change attitudes, decrease unhealthy habits, improve the management of chronic conditions and generally increase health related quality of life [15].

Health coaching can improve treatment adherence in chronic patients [16], is effective towards glycemic control and diabetes [17], oncological pain, self-management of pain [18], moderate weight loss and the improvement of healthy habits that result in weight loss and the promotion of a healthy diet and physical activity [19].

Several research protocols currently include coaching-based interventions to promote healthy lifestyles in elderly people at moderate risk of suffering from cardiovascular conditions, diabetes and depression, and also for patients on low incomes and poorly-controlled diabetes, hypertension or hyperlipidemia. Coaching is conducted by a medical assistant as a health coach and with telephone support [20]. These interventions can be carried out by different health professionals and even by people suffering from the same condition (peer health coaching); in this last instance, these patients must receive coaching training. In the study of Thom and colleagues, the researchers themselves were the coaches [17], whereas in Thomas et al. the trainer was a psychologist [18].

No studies on the application of health coaching to osteoarthritis of the knee have been published to date. Moreover and to our knowledge, clinical guidelines for osteoarthritis of the knee are not implemented in primary care when the treatment of choice is non-pharmacological. Since most guidelines recommend non-pharmacological treatment for pain self-management, weight loss and increased physical activity [1,2,7,8], our study aims to provide evidence on the effectiveness of health coaching on KO by developing a complex intervention that will focus on the patient and will encourage participation, to promote the patients’ knowledge of their condition and to facilitate the achievement of their therapeutic goals in accordance with their own resources. Telephone support will ensure that the patient manages the osteoarthritis with the highest possible independence and quality of life [7].

This study aims to implement a flexible, complex intervention adapted to the people with osteoarthritis and that is feasible, effective and sustainable in primary care centres. The intervention aims to promote healthy behaviour, decrease unhealthy habits and improve quality of life and the control of osteoarthritis. Ultimately, it should encourage a healthy and active ageing process by preventing also other prevalent diseases. Indeed, health promotion behaviours contribute to a less dependent and a more satisfactory older age.

In line with the WHO recommendations for active ageing [21], to remain socially and mentally active the coaching intervention of our study will encourage the use of the patient’s own strategies to: 1. promote healthy habits and physical exercise to reduce risk factors of disease; 2. promote cognitive vitality; 3. promote a positive approach to problems and emotions; 4. promote social participation [15].

If the study proves the cost-effectiveness and cost-utility of the intervention, a local adaptation could be incorporated into the clinical guidelines and implemented in primary care centres. In our study the health coach is a psychologist who will also train primary care professionals to integrate coaching in everyday clinical practice.

## Objectives

### Main objective

To analyze effectiveness, cost-effectiveness and cost-utility of an intervention based on health coaching and telephone support on quality of life, pain, overweight/obesity and physical activity in patients from Primary Health Care Centres (PHCC) of the Barcelona province suffering from osteoarthritis of the knee, compared with usual care.

### Secondary objectives

To identify the barriers and facilitators of an intervention based on health coaching with a qualitative study that includes individual interviews to patients suffering from osteoarthritis and group interviews with primary care professionals, with the aim to design an intervention adapted to primary care patients and professionals. Based on the experience and opinion of participating patients and professionals, to evaluate the acceptability and feasibility of the intervention.

## Methods/Design

The Medical Research Council has established a methodology for this type of complex interventions that consists in several phases that can be repeatedly implemented and that use qualitative and quantitative methods [14]. Our study comprises three phases:

### Phase 1: modeling and operationalization of the intervention

#### Qualitative study

The aim of the phase is to identify the key factors that can influence the development of the intervention. It will identify the barriers and facilitators of the health coaching intervention on KO for patients and health professionals. The participants in this study will be adults with osteoarthritis of the knee and the primary care professionals responsible for treating this condition. In this phase we will specify the different components of the intervention and determine the factors related to patients, professionals and other that facilitate or restrict the intervention (acceptability, adequacy, feasibility, integration within other programmes, location, schedule and duration).

The qualitative study will use a socioconstructivist perspective and theoretical sampling. Ten semi-structured interviews will be conducted with patients suffering from osteoarthritis to identify their beliefs, knowledge and perceptions on the components and conceptual model of the intervention.

We aim to reach discourse saturation through interviews to men and women of different ages and literacy levels. The four discussion groups will include 8–12 primary care professionals working with patients with osteoarthritis to identify their attitudes, opinions, motivation and to verify their competence on coaching, knee osteoarthritis and information and communication technology (ICT) [22].

#### Operationalization of the intervention

The results of the qualitative study will provide information towards the operationalization of the intervention, so that it adapts to the needs and reality of the PHCCs patients and professionals. An informative session will be held in the participating PHCCs to explain the results of the qualitative study; the objectives and characteristics of the study and the intervention and recruitment strategies will be explained to the professionals. Recruitment will be coordinated by a research team member together with the designated professional of the PHCC.

#### Analysis of qualitative data

A sociological discourse analysis based on Hodges’ criteria will be carried out [23]. Masked, anonymized literal transcriptions of the recorded sessions will be produced. After consecutive readings of the transcriptions and the formulation of preanalytical intuitions, we will analyse the social status of patients and professionals. Next, we will analyse the discursive fractions, determined by their positioning in relation to the subject discussed (discursive positioning). Then, we will generate a global interpretation of the text in relation to the objectives of the research to organize the whole discourse and to link it to the context (symbolic configuration). Next, we will carry out the internalist analysis of the text looking for semantic attractors and associative chains (semantic configuration) with the assistance of the Atlas-Ti programme. Finally, the discourse will be reconstructed and the analysis will be triangulated between the members of the research team.

#### Applicability of results

The results of Phase 1 will provide information on barriers and facilitators for the design of a flexible intervention adapted to the needs of primary care patients and professionals.

### Phase 2: study of effectiveness, cost-effectiveness and cost-utility

#### Design

Cluster randomized clinical trial. The PHCC will be the unit of randomization.

#### Study setting

18 PHCCs of the province of Barcelona that agree to participate (9 PHCCs per study group). The PHCCs will be randomized into the complex, multidisciplinary intervention (health coaching with telephone support + usual care in PC) or the control group (usual care in PC).

#### Participants

360 participants, 180 per group.

#### Inclusion criteria

Level 1. Inclusion criteria to be determined by the physician: primary care patients with clinical and radiological diagnosis of knee osteoarthritis in the Kellgren-Lawrence stages 1–3 [24]; to be able to read and write; to have a mobile phone; to be able to go to the PHCC; and to agree to participate. Level 2. Inclusion criteria to be determined by the psychologist: patients that deliver the “commitment folder” within the period agreed. This report will measure the stage of change [13] of the patient [11].

#### Exclusion criteria

The following patients will be excluded: patients with knee osteoarthritis in the Kellgren-Lawrence stages 0 and 4; patients with rheumatoid arthritis, fibromyalgia and other systemic rheumatological conditions; patients on a waiting list for orthoses; patients admitted during the past three months for cardiovascular diseases; patients suffering from Parkinson disease, Paget’s disease, cognitive deterioration, metastatic cancer, severe mental diseases and personality disorders; women pregnant or planning a pregnancy.

### Intervention design

#### Intervention Group (IG)

The intervention programme is divided in two phases: the intensive phase, which lasts 20 hours distributed along one month (Table 1); and the follow up phase. The results of Phase 1 of the current study will determine the periodicity and other components (described in the phase 1) of the intensive phase.

**Table 1:** **Intervention programme: intensive phase**

**Duration**	**Goals**	**Contents**	**Components**
2 hours	• Presentation	**About Health Coaching:**	1. Motivation Techniques
	• Sharing and Explaining Concepts	1. What is health coaching?	2. Group Development Techniques
	• Motivate participants	2. Who can benefit ?	3. Evidence-based Information
	• Agree conditions of intervention	3. The health coaching process	
		a. General outline	
		b. Confidentiality (with reference to ethical code)	
		4. What is it useful for?	
		**Knee Osteoarthritis:**	
		1. What is it?	
		2. How can it be treated?	
		Tasks	
		Agreement on conditions of intervention	
12 hours	• Increase physical activity	1. Review of Tasks	1. Motivation Techniques
	*Increase healthy diet	2. Goal Setting	2. Group Development Techniques
	*Increase self-management of pain techniques and strategies	3. Analyze current situation	3. Evidence-based Information
		4. Options	
		5. Action Plan	
		6. Creation of guideline for self-monitoring (goal achievement process)	
		7. Creation of guideline for self-evaluation of action plan (goal achievement)	
6 hours	• Follow-up of Action Plan and Evaluation of Results	1. Provide the patient with strategies and techniques to follow the action plan through	
		2. Review Action Plan	
		3. Review Evaluation of Results	
		4. Evaluation of Results	
		5. Prevention of Relapse	
**Intervention Programme-follow-up phase**			
**Frequency**	**Goals**	**Contents**	**Components**
Once monthly	Maintain Motivation Weight Control	Positive Reinforcement Empower and Train Laser Questions	1. Motivation Techniques
			2. Group Development Techniques
			3. Evidence-based Information

A psychologist coach is in charge of the intensive phase, which includes motivational, coaching psychology and group development techniques, and evidence based information. Four pillars (mindful presence, authentic communication, self-awareness and safe place) [25] and seven elements that determine behaviour change (self-regulation, skills, self-efficacy, expected outcome, intention, context and regulations) [11] are the cornerstone of health coaching.

#### Allocation of time

1. Two hours for the following objectives: presentation of the programme, merge the prior knowledge of participants, motivate them and agree on the conditions of the intervention. The following aspects will be discussed: what is health coaching psychology, who can benefit from health coaching, how it works, how it is implemented, confidentiality and aims of the intervention. The treatment and causes of knee osteoarthritis will be also discussed.
2. Twelve hours for the following objectives: scale up physical activity, improve nutrition and increase strategies for the self-management of pain. The following aspects will be discussed: definition of goals, analysis of the current situation, options, action plan and production of individual guidelines for the self-assessment of goal attainment and for the self-assessment of the action plan.
3. Six hours for the following objectives: follow up of the action plan and assessment of results. The following aspects will be discussed: techniques and strategies to follow through the action plan, review of the action plan, review of the assessment of results, assessment of results and prevention of relapse.

The follow up will take place in the primary care centre of the participant, with their own GP and nurse. The nurse and GP will receive training to sustain motivation and weight control. Specifically, they will be trained on positive feedback, patient empowerment and laser questions with motivation and coaching psychology techniques and evidence based information. The pillars and elements of behaviour change will be the same as in the intensive phase. These individual sessions will be conducted monthly and they will last as much as any other follow up visit.

Compliance in relation to the group sessions will be assessed by registering the number of group sessions attended. To ensure patients’ adherence to the group sessions, they will be able to choose between morning or afternoon sessions. One week prior to the group session, they will receive a phone call to remind them the date, time and place of the session and on the day before they will receive an SMS reminder.

#### Control Group (CG)

The participants allocated to the CG will follow usual care based on recommendations and brief advice by the primary care physician and/or nurse [1,7,8], as recommended by the clinical guidelines. These recommendations to control pain, maintain functionality and prevent progression of disease include: weight control, correct body posture, thermotherapy, adherence to treatment, physical exercise, rest and orthosis.

#### Recruitment

**Figure 1:**
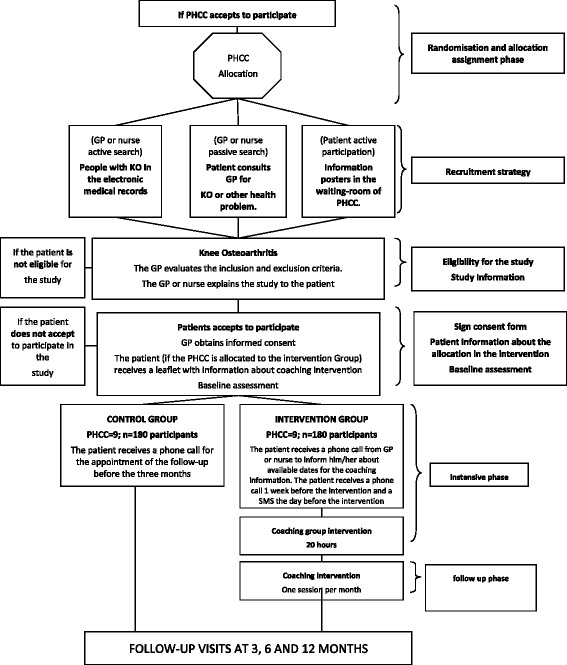
**Flow chart (Phase 2).** Notes: OK = Osteoarthritis Knee; GP = general practitioner; PHCC = Primary Health Care Centres.

In addition to considering the results of Phase 1, recruitment will take place through: 1. Patients who go to the doctor for knee osteoarthritis or other health problems; 2. People with a diagnosis of knee osteoarthritis in the electronic medical records; 3. Information posters in the waiting rooms of the PHCCs. If the patient agrees to participate, the physician and/or nurse will give him/her a “commitment folder” and the informed consent form. The patient must study the documentation and bring it back to the PHCC within two weeks (See Figure 1).

#### Outcomes

The main outcome variable is quality of life as measured by the WOMAC index [26]. Secondary outcomes are: pain [27], weight, physical activity [28] and nutrition [29].

#### Other variables

The main independent variable is the intervention arm: coaching group intervention or usual care.

Socio-demographic, clinical and cost variables will be recorded. Table 2 shows the study variables and the measurement instruments, validated in Spanish and for primary care.

**Table 2:** **Data collection, information sources and follow-up**

**Variables**					**Data collection**				
					**Baseline**	*Follow up from recruitment			
**Clinical dependent variables**	**Measurement instrument**	**Type of response**	**Scoring (total and per item)**	**Range**	**Before intensive phase**	**After intensive phase**	**3 months**	**6 months**	**12 months**
Qual ity of Life	WOMAC (Batl le, 1999)	Likert (0=none to 4=very much)	TOTAL=24 17= functional l imitation 5=pain 2= sti ffness	0-96	X	X	X	X	X
Pain	ICOAP (Mai l lafert, 2009)	Likert (0=none to 4=very much)	TOTAL = 11 5= constant pain 6= intermittent pain	0-44	X	X	X	X	X
Weight (in Kg.)*	Scale at the PCC	Quantitative (kg.)	-	0 - ∞	X	X	X	X	X
Physical Activity	IPAQ (Puig, 2012)	Duration + Frequency of Moderate to Intense	TOTAL= 7	0 - 3000 MET	X	X	X	X	X
Physical Activity*	Pedometer	Quantitative	-	0 - ∞					
Nutrition	PREDIMED (Mediterranean Diet Adherence Questionnaire	Dichotomous (YES= 1 point NO= 0 points)	TOTAL = 14 12= frequency of consumption of foods 2= Mediterranean food habits	0-14	X	X	X	X	X
Other Variables									
Socio-demographic									
Age, gender, educational level, work status, number of children	Ad-hoc Questionnaire	Nominal	-	-	-	X			
Clinical				-					
Drug Prescription	Ad-hoc Questionnaire	Nominal	1	-	X	X	X	X	X
Diagnostic tests	Ad-hoc Questionnaire	Nominal	1	-	X				X
Waist Circumference	Ad-hoc Questionnaire	Quantitative (cm.)	1	-	X	X	X	X	X
Height	Ad-hoc Questionnaire	Quantitative (cm.)	1	-	X				X
Comorbidities	Ad-hoc Questionnaire	Nominal	1	-	X	X	X	X	X
Duration of Disease in Years	Ad-hoc Questionnaire	Quantitative (years)	1	-	X				
Duration Knee Osteoarthritis in	Ad-hoc Questionnaire	Quantitative (years)	1	-	X				
Costs-related			-						
Direct Medical			-						
Cost of coaching in osteoarthrosis	Ad-hoc Questionnaire	Quantitative (euros)	-	-	X	X	X	X	X
Cost of visits to the GP	Ad-hoc Questionnaire	Quantitative (euros)	-	-	X	X	X	X	X
Cost of visits to the nurse	Ad-hoc Questionnaire	Quantitative (euros)	-	-	X	X	X	X	X
Cost of visits to the physiotherapist	Ad-hoc Questionnaire	Quantitative (euros)	-	-	X	X	X	X	X
Cost of visits to special ist MD	Ad-hoc Questionnaire	Quantitative (euros)	-	-	X	X	X	X	X
Cost of Medical Tests	Ad-hoc Questionnaire	Quantitative (euros)	-	-	X	X	X	X	X
Cost of Pharmacological	Ad-hoc Questionnaire	Quantitative (euros)	-	-	X	X	X	X	X
Operating Costs of the PCC	Ad-hoc Questionnaire	Quantitative (euros)	-	-	X	X	X	X	X
Cost of Disposable Medical Equipment	Ad-hoc Questionnaire	Quantitative (euros)	-	-	X	X	X	X	X
Cost of transport (ambulance)	Ad-hoc Questionnaire	Quantitative (euros)	-		X	X	X	X	X
Cost of Home Assistance	Ad-hoc Questionnaire	Quantitative (euros)	-		X	X	X	X	X
Indirect Medical			-						
Costs due to loss of productivity (days of sick leave)	Ad-hoc Questionnaire	Uantitative (euro	-		X	X	X	X	X

* Monthly.

#### Data collection, information sources and follow-up

All variables will be measured individual level. All participants will be invited to attend the PHCC for outcome assessments. The variables will be measured in both groups between 5 and 10 days before the intervention, immediately after the intensive phase (1 month) and after 3, 6 and 12 months of recruitment (Table 2). During the first interview we will measure the height (in meters). The same scale of the PHCC will be used to measure weight. During the follow up phase of the study, the nurse will measure weight and record physical activity for the intervention group. Participants from both groups will receive reminders before follow up visits. For each assessment, one of the study researchers will call up to three times during the day to make the appointment. During the visit, he/she will fill out the questionnaires by interviewing the participant; other clinical data will be extracted from the electronic medical records by the general practitioner or nurse. Empathic communication with study participants will be sustained during all phases. Two independent investigators will enter the data in a centralized database; data quality will be assessed.

#### Randomization

To avoid contamination between study groups, the PHCC will be the randomization unit. The PHCCs that agree to participate will be allocated either to the IG or the CG according to a random sequence generated by a computer programme. The allocation of the PHCC to the study groups will be carried out by an independent researcher. The patients that fulfil the inclusion criteria will be allocated to the treatment group of their PHCC.

#### Blinding

To avoid bias, the informed consent of participants will be obtained before the disclosure of the randomization results. Due to the characteristics of the intervention patients, physicians and nurses will know their group allocation. The analyst will not know the allocation group of the patient.

#### Sample size

Sample size calculation is based on the minimal significant change in the clinical parameters and in the impact of the osteoarthritis using the WOMAC index [30]. In order to achieve a power of 80% (beta: 0.2) and a significance level (alpha) of 0.05 for a two-tailed comparison, 124 participants will have to be recruited in each group to detect differences equal or higher than 5 units in the Womac index. An estimated standard deviation of 14, a correlation between the first and second measurement of 0.6 and 20% loss to follow-up have been assumed for these calculations. To take into account the randomization by PHCC, we consider a design effect of 1.45 with a mean number of patients per intervention group of 10 and an intraclass correlation coefficient of 0.05 [31]. The required sample size has been estimated at 180 patients per group: 9 primary care teams with 20 patients each will be recruited for each study group. The statistical package GRANMO v7.12 (IMIM, BCN, Spain) was used for sample size calculation.

#### Statistical analysis

Data will be analyzed on an intention-to-treat basis following CONSORT CLUSTER criteria to avoid bias by incomplete datasets. Missing values will be replaced by multiple imputation methods. The non-response bias will be monitored and evaluated during follow up. According to the distribution of the variables, descriptive analysis will be carried out using the mean (standard deviation), median (interquartile range) or frequency (percentage).

For comparisons within and between the intervention and control groups we will use Student t-tests for independent and paired data, McNemar, Chi-Square, Fisher’s Exact test, analysis of variance and the corresponding non-parametric tests when appropriate.

To evaluate the effectiveness of the intervention between both groups during follow up, the change in the intervention group minus the change in the control group, as well as the standardized effect size (SES) will be calculated. SES will be calculated as the mean difference between both groups divided by the standard deviation of the control group. To detect differences within each group, the difference between the means at baseline and at each follow up time and the effect size or standardized response mean (SRM) following Kazis’method will be calculated [32]. The SRM will be calculated as the mean change divided by the standard deviation of the change. To evaluate SRM and SES we will use Cohen’s rule, which classifies effect size as small (0.2-0.5), medium (0.5-0.8) and large (>0.8).

We examined the effects of intervention over all time points using mixed-effects models on repeated measures.

Multilevel ordered logistic regression will be used for the response variable “physical activity”, in which patients are categorized in three levels: low: <600 METs. min/week; moderate: > = 600 and <1500 METs min/week; high: between 1500 and 3000 METs min/week.

The PHCC will be considered a random effect. The intraclass correlation coefficient will be calculated to determine which percentage of variability in the response is due to the PHCCs. The final models will be adjusted for potential confounders and relevant clinical variables. Interactions and colinearity will be evaluated [33]. Significance level of the model has been set at 5%. The statistical packages SPSS for Windows, v.21 (IBM SPSS Inc., Chicago, IL) and Stata/SE v.12 for Windows (StataCorp. LP, TX) will be used for statistical analysis.

#### Analyses of the economic evaluation

A descriptive analysis of costs and use of resources will be carried out for each study group and these groups will be compared (Table 2).

The time horizon will be of 12 months. The discount rate will be 3%. The incremental cost-effectiveness ratio will be calculated as the difference in the mean costs between both groups divided by the difference between effects of both groups. Health utilities will be calculated through the WOMAC index adjusted by time. The incremental cost-utility ratio will be calculated dividing the difference in mean total costs in both groups by the differences of QALYs of both groups. Acceptability curves will be calculated to determine if the intervention is cost-effective compared with standard clinical practice. An analysis of sensitivity with different discount rates and costs will be carried out to verify the robustness of results.

#### Applicability of results

This phase aims to show the effectiveness and cost-effectiveness of the health coaching intervention on quality of life improvement, reduction of pain and overweight and increase of physical activity in people suffering from KO.

### Phase 3: evaluation of the intervention programme

A qualitative evaluation of the intervention will take place after Phase 2 to determine which aspects can be improved and the opinions and experiences of participating professionals and patients.

The participants of the second qualitative study (Phase 3) will be adults with osteoarthritis of the knee and primary care professionals that participated in the coaching intervention arm.

Ten semi-structured interviews will be conducted with patients in the IG to check if the intervention has improved their quality of life, reduced pain, contributed to weight loss or increased physical activity. These patients will also be asked about the suitability of times, adaptability and clarity of the intervention contents. Plurality of discourse will be achieved by interviewing participants of both genders, different ages and educational levels. Four discussion groups of 8–12 primary care professionals involved in osteoarthritis care will be created to analyze attitudes, opinions on the intervention and adaptability to standard clinical practice in primary care. The analytical plan will be as in Phase 1.

#### Applicability of results

This phase aims to evaluate the acceptability and feasibility of the intervention according to the opinions and experiences of patients and professionals that participated in Phase 2.

### Ethics

The study will be conducted according to the tenets established by the Declaration of Helsinki and Tokyo. The study protocol has been approved by the Clinical Research Ethics Committee of the Primary Health Care University Research Institute-IDIAP Jordi Gol.

## Discussion

The promotion of active, healthy ageing is a challenge generated by the increase in life expectancy in most developed countries. To this end, effective strategies are required to assist people in the modification of their lifestyles. People with established chronic conditions or at risk of developing them need to change behaviors to improve their health, quality of life and to make a better use of health services.

Clinical practice of knee osteoarthritis in primary care is not standardized. Most guidelines agree on the need to start with a non-pharmacological approach and to focus on lifestyle changes that will also prevent adverse effects of treatments and reduce costs. Since health coaching has been effective in lifestyle changes, our study aims to influence the lifestyle of patients suffering from knee osteoarthritis, most of them over the age of 60 and with comorbidities.

In the introduction we mentioned Argyris’ Action Theory. Argyris differentiates between the chosen theory, the theory we verbalize when we are asked about the motivations and approaches to our tasks, and the Theory-In-Use inferred from the observation of our actions; these theories do not always coincide. People do not always express what they actually do, the main reason being the lack of consciousness on how we really act in a situation. If we focus on explaining the patients what we must do (education) it is very likely that we change the words of the chosen theory without changing what we actually do [12].

Our study aims to empower participants after they have observed their actual behaviour and understood its undesired consequences. Next, the contexts where that behaviour has been learned will be analyzed (meta-reflection). Finally, new practices to replace past behaviours will be rehearsed. Accordingly, during the development of the study professionals and patients will reflect on their own practices and behaviours. This will directly impact on patients by enhancing their quality of life and promoting healthier lifestyles and on professionals by improving the results of their practice. It will also generate novel communication strategies between patients and professionals.

This study contains the following limitations: (1) the participants are patients in the PHCCs, which could affect the external validity of the results. However, our health system has universal coverage and over 70% of the population attends yearly the PHCCs; (2) patients with knee osteoarthritis present comorbidities and generally visit the PHCC more often; (3) despite the high prevalence of knee osteoarthritis, clinical practice is highly heterogeneous and the clinical guidelines are barely followed. However, the variability in clinical practice should affect equally the intervention and control groups and the multilevel analysis will take into account and quantify this variability.

This study aims to evaluate the effectiveness and cost-effectiveness of a complex intervention conducted in primary care centres through a Health Coaching Programme on patients with knee osteoarthritis, most of whom are over 60 years of age. The improvements on society and on the patients’ quality of life are in accordance with the WHO tenets for active ageing. Consequently, with this intervention we aim to: 1. achieve a significant improvement of health; 2. assist in changing and maintaining healthy lifestyles and improve the clinical management of patients with knee osteoarthritis; 3. promote active ageing; 4. prevent other conditions thanks to lifestyle changes; 5. provide strategies to remain physically active; 6. provide strategies to remain socially active; 7. provide strategies to remain mentally active; 8. if the study shows the cost-effectiveness and cost-utility of the intervention the results could be transferred to the clinical guidelines and implemented in the primary care centres; 9. allow the professionals and the patients to voice their opinions and their experience of the study; 10. maintain healthy lifestyles through asset mapping; 11. inform primary care professionals via Newsletter; 12. disseminate the results in international journals; 13. disseminate the results through a manual for health professionals; 14. disseminate the results to the general public; 15. disseminate the results in national and international meetings.

## Abbreviations

WOMAC: Western ontario mcmaster universities osteoarthritis index
QALYs: Quality adjusted life years
KO: Osteoarthritis of the knee
PC: Primary care
PHCC: Primary health care centre
MRC: Medical research council
QoL: Quality of life
ICT: Information and communication technology
IG: Intervention group
CG: Control group
SES: Standardized effect size
SRM: Standardized response mean
MET: Metabolic equivalent
